# Cerebellum regulating cerebral functional cortex through multiple pathways in complete thoracolumbar spinal cord injury

**DOI:** 10.3389/fnins.2022.914549

**Published:** 2022-07-29

**Authors:** Weimin Zheng, Ling Wang, Beining Yang, Qian Chen, Yongsheng Hu, Jubao Du, Xuejing Li, Xin Chen, Wen Qin, Kuncheng Li, Jie Lu, Nan Chen

**Affiliations:** ^1^Department of Radiology and Nuclear Medicine, Xuanwu Hospital, Capital Medical University, Beijing, China; ^2^Beijing Key Laboratory of Magnetic Resonance Imaging and Brain Informatics, Beijing, China; ^3^Department of Radiology, Beijing Friendship Hospital, Capital Medical University, Beijing, China; ^4^Department of Functional Neurosurgery, Xuanwu Hospital, Capital Medical University, Beijing, China; ^5^Department of Rehabilitation Medicine, Xuanwu Hospital, Capital Medical University, Beijing, China; ^6^Department of Radiology, China Rehabilitation Research Center, Beijing, China; ^7^Department of Radiology, Tianjin Medical University General Hospital, Tianjin, China

**Keywords:** complete thoracolumbar spinal cord injury, cerebellar subregions, cerebral cortex, functional connectivity, resting state functional magnetic resonance imaging

## Abstract

The previous studies have found significant brain structural and functional changes in cerebral regions after spinal cord injury (SCI), but few studies have explored the cerebellar–cerebral circuit changes in SCI. This study aims to study the brain structural changes of cerebellar subregions and its functional connectivity (FC) changes with cerebrum in complete thoracolumbar SCI (CTSCI), and screen out the regions that play relatively important roles in affecting sensorimotor function. Eighteen CTSCI patients and 18 age- and gender-matched healthy controls (HCs) were recruited. Voxel-based morphometry (VBM) was used to characterize the brain structural changes of cerebellar subregions [from the Anatomical Automatic Labeling (AAL116)], seed-based FC was used to evaluate the cerebellar–cerebral FC changes and support vector machine (SVM) analysis was used to search for sensitive imaging indicators. CTSCI patients showed slightly structural atrophy in vermis_3 (*p* = 0.046) and significantly decreased FC between cerebellum and cerebral sensorimotor-, visual-, cognitive-, and auditory-related regions (cluster-level FWE correction with *p* < 0.05). Additionally, SVM weight analysis showed that FC values between vermis_10 and right fusiform gyrus had the greatest weight in functional changes of CTSCI. In conclusion, different degrees of structural and functional changes occurred in each subregion of cerebellum following CTSCI, and FC change between vermis_10 and right fusiform gyrus plays the most important role in dysfunction and may become an important neural network index of rehabilitation therapy.

## Introduction

The previous studies on brain structural and functional alteration after spinal cord injury (SCI) have mostly focused on the cerebral changes (Jurkiewicz et al., 2006; Wrigley et al., 2009; Freund et al., 2011, 2013; Zheng et al., 2017a; Chen et al., 2018; Li et al., 2020), although some studies have also found that the structural and functional changes occurred in part of cerebellar subregions following SCI (Li et al., 2020; Lei and Perez, 2021), little attention was paid to the pathway and role of the cerebellum in regulating the function of the cerebral cortex after SCI. Clarifying the structural and functional changes of each cerebellar subregion, and further screening out the regions that play relatively important roles in affecting sensorimotor function, would be helpful to deeply understand the reorganization of brain function after SCI, and may have important theoretical guiding for finding new key neural network targets in rehabilitation therapy such as transcranial direct current stimulation (tDCS) or brain–computer interface (BCI).

Cerebellum contains more than half of the brain neurons, though it only accounts for about 10% of the brain’s capacity. In addition to the coordinating movement, the human cerebellum is also related to cognition, emotions function, and behaviors dominated by different subregions of the cerebellum (Baillieux et al., 2008). At present, animal experimental research has showed that SCI can lead to loss of neurons in the cerebello-spinal fasciculus, resulting in changes in cerebellar circuits (Visavadiya and Springer, 2016). In addition, the number and synaptic contacts of purkinje cells, which is closely related to the cerebellar dependent motor learning, will be decreased following SCI (Visavadiya and Springer, 2016; Hassanzadeh et al., 2018). In humans, axons carrying proprioceptive signals from the cervical spinal cord terminates in cerebellar lobules (Jang and Kwon, 2014), are often impaired following SCI and lead to cerebellar atrophy (Ozdemir and Perez, 2017). Therefore, changes in the cerebellum play important roles in SCI patients, and the study of the cerebellum microstructure is crucial for understanding the mechanism of SCI. There is a series of parallel circuits between the cerebellum and the cerebral cortex, and information can be projected from certain areas of the cerebral cortex to the cerebellum regions responsible for receiving information, which provides the structural basis for the cerebellum to participate in various higher functions such as sensorimotor, cognition, vision, and so on. The cerebellum is divided into several subregions, each of which has obvious functional heterogeneity. At present, the role of cerebellar subregion in affective disorders such as AD and schizophrenia has been well-studied (Wang et al., 2016; Zheng et al., 2017b). However, the study on the cerebellar–cerebral functional connectivity (FC) changes following SCI has not been reported yet, it is meaningful to reveal the neural mechanism of SCI from the perspective of cerebellum.

In SCI patients, the FC intensity between each cerebellar subregion and different cerebral cortex may be different. Therefore, finding out the brain regions that play relatively important roles in SCI through machine learning classification algorithm can better clarify the main pathway that cerebellum affects the cerebral cortex, which is helpful to finding new neural circuits or therapeutic targets for rehabilitation. Support vector machine (SVM) (Cortes and Vapnik, 1995) is one of the most commonly used machine learning algorithms in neuroimaging data analysis. Based on the feature that SVM can work well in situations where the number of samples is far less than the number of features, it is very suitable for typical neuroimaging data.

In this current study, we will explore the structural changes of cerebellar subregions and the FC changes between cerebellar subregions and cerebral cortex in patients with complete thoracolumbar SCI (CTSCI), and further screen out the regions that play relatively important roles in affecting sensorimotor function by SVM analysis.

## Patients and methods

### Participants

Eighteen right-handed complete thoracolumbar SCI (CTSCI) patients (28–67 years of age; 16 men) and 18 age-, gender-matched right-handed healthy controls (HCs) (27–64 years of age; 16 men), participated in this study. Each of the subjects provided a written informed consent, and is in accordance with a protocol approved by the Medical Research Ethics Committee of Xuanwu Hospital, and the Declaration of Helsinki. The extent of motor and sensory impairment in SCI patients were assessed by qualified clinician (by Dr. Jubao Du who is a rehabilitation doctor with extensive clinical experience) using the classification scale of the American Spinal Cord Injury Association (ASIA) (Marino et al., 2003). A full neurological examination was performed in order to exclude accompanying neurological disorders of the peripheral and central nervous systems. Clinical and demographic data from all participants are shown in Table 1.

**TABLE 1:** Clinical data of 18 complete thoracolumbar spinal cord injury (CTSCI) patients and 18 healthy controls (HCs).

	CTSCI group (18)	HC group (18)	*P*
Age	48.5 ± 9.86	45.17 ± 10.35	0.963
Gender (man/woman)	16/2	16/2	1.000
Etiology	15 trauma/1 surgery/2 fall	−	−
Level of lesion	T6-L3	−	−
ASIA	18A	−	−
Time since injury (months)	1-348	−	−
Motor scores (0–100)	50.72 ± 1.88	−	−
Pinprick	72.78 ± 9.07	−	−
Light touch	72.83 ± 9.11	−	−
Sensory scores (0–224)	145.61 ± 18.18	−	−
VAS	7.56 ± 2.19	−	−

The level of lesion refers to the neurological level. ASIA impairment scale: A: complete—no sensory or motor function is preserved in sacral segments S4–S5; B, incomplete—sensory but not motor function is preserved below the neurological level and extends through sacral segments S4-S5; C: incomplete—motor function is preserved below the neurological level, and more than half of the keymuscles below the neurological level have a muscle grade of <3; D: incomplete—motor function is preserved below the neurological level, and at least half of the key muscles below the neurological level have a muscle grade of >3. Sensory score: sum of segmental light touch and pinprick classifications. CTSCI, complete thoracolumbar spinal cord injury; HC, healthy control; M, month; ASIA, American Spinal Injury Association; VAS, visual analog scale.

### Magnetic resonance imaging data acquisition

The MRI images were obtained from the 3.0-T MRI system (Trio Tim, Siemens, Erlangen, Germany) with a 12-channel phased-array head coil. The parameters of high-resolution three-dimensional (3D) structural T1-weighted images were as follows: repetition time (TR)/echo time (TE)/inversion time (TI) = 1,800 ms/2.13 ms/1,100 ms; flip angle (FA) = 9°; number of slices = 192; slice thickness = 1 mm; field of view (FOV) = 256 mm^2^ × 256 mm^2^, matrix = 256 × 256, isotropic voxel size = 1 mm^3^ × 1 mm^3^ × 1 mm^3^. The parameters of resting-state functional images were as follows: 35 axial slices with a slice thickness = 3 mm, inter-slice gap = 1 mm, TR/TE = 2,000 ms/30 ms; FA = 9°; FOV = 220 mm^2^ × 220 mm^2^, matrix = 64 × 64, isotropic voxel size = 3.4 mm^3^ × 3.4 mm^3^ × 3.0 mm^3^, the acquisition time of resting-state functional images was 6.08 min with 180 volumes.

### Structural data preprocessing and voxel-based morphometry analysis

The structural data preprocessing was performed using Statistical Parametric Mapping (SPM) software^1^ implemented in MATLAB 2018b (Math Works, Natick, MA, United States). The preprocessing steps are as follows: first, all structural magnetic resonance images were manually reoriented to place the anterior commissure at the origin. Then, based on the unified standard segmentation option in SPM, images were segmented into gray matter (GM), white matter (WM), and cerebrospinal fluid (CSF) areas. Additionally, individual GM components were normalized into the standard Montreal Neurological Institute (MNI) space using the Diffeomorphic Anatomical Registration through Exponentiated Lie algebra (DARTEL) algorithm (Ashburner, 2007). The normalized GM component was modulated to generate the relative GM volume (GMV) multiplied by the non-linear part of the deformation field at the DARTEL step. The resulting GMV images were then smoothed with an 8-mm full-width at half-maximum (FWHM) Gaussian kernel. Finally, the GMV of each subject in each cerebellar subregion was calculated using REST V1.82 software (in MATLAB) for the subsequent statistical analysis.

### Functional data preprocessing and functional connectivity analysis

The resting state functional magnetic resonance imaging (rs-fMRI) data preprocessing was performed using the Data Processing Assistant for Resting-State fMRI (DPARSF) (Chao-Gan and Yu-Feng, 2010) with the following steps: data conversion, removal of the first 10 volumes, slice timing and head motion correction (data with maximum translation exceeding 2.5 mm and maximum rotation exceeding 2.5° was excluded), normalization (realigned volumes were spatially standardized into the MNI space using the echo-planar-imaging (EPI) template and images were resampled into a voxel size of 3 mm^3^ × 3 mm^3^ × 3 mm^3^), smooth (with a Gaussian kernel of 4 mm FWHM), and regression (twenty-four motion parameters, their first time derivations, WM, and CSF). All the cerebellar subregions were defined as regions of interests (ROIs) from the Anatomical Automatic Labeling116 (AAL116) template (Tzourio-Mazoyer et al., 2002), among which AAL (1-90) belong to the cerebrum, and the remaining 26 regions (AAL91-116) belong to the cerebellum. After band-pass filtering (0.01–0.08 Hz), a reference time series for each seed was extracted by averaging the time series of voxels within each ROI, and correlation analysis was performed between the seed region and the remaining voxels in the whole brain. To improve normality, the resulting *r* values were converted by Fisher’s r-to-z transformation (Liu et al., 2017).

### Statistical analyses

To assess between-group (CTSCI patients *vs*. HCs) differences of structural changes in the cerebellar subregions, independent-samples *t*-test was performed, with age, gender, and injury time as covariates (*p* < 0.05, SPSS20.0).

Then, two-sample *t*-test was performed with age, gender, and injury time as covariates to analysis the FC changes between CTSCI patients and HCs when all the cerebellar subregions were defined as ROIs using SPM. The significance threshold was set to Family Wise Error (FWE) correction (cluster-wise, *p* < 0.05).

Additionally, to explore the relationships between the clinical features (ASIA scores and time since SCI) and the structural changes and FCs of cerebellum in CTSCI patients, a partial correlation analysis was performed with age and gender being used as nuisance covariates (*p* < 0.05, SPSS20.0).

Finally, to evaluate the weight of abnormal cerebellar functional features in CTSCI, we adopted the SVM (Chang and Lin, 2011) method in MVPANI (Peng et al., 2020) though the MATLAB 2018b. During the SVM training, a 10-fold leave-one-out cross-validation method was used to train our model. We then calculated the models accuracy, sensitivity, specificity, and other indicators on the results. We repeated this process 10 times and calculated the mean value of these experimental results.

## Results

### Demographic and clinical characteristics

Eighteen patients with CTSCI patients and 18 age- and gender-matched HCs were recruited in this study. Demographic and clinical data for all the participants were shown in Table 1. No statistically significant differences were found between CTSCI patients and HCs in age (*p* = 0.963) and gender (*p* = 1.000).

### Brain structural and functional changes of cerebellar subregions in complete thoracolumbar spinal cord injury patients

**FIGURE 1:**
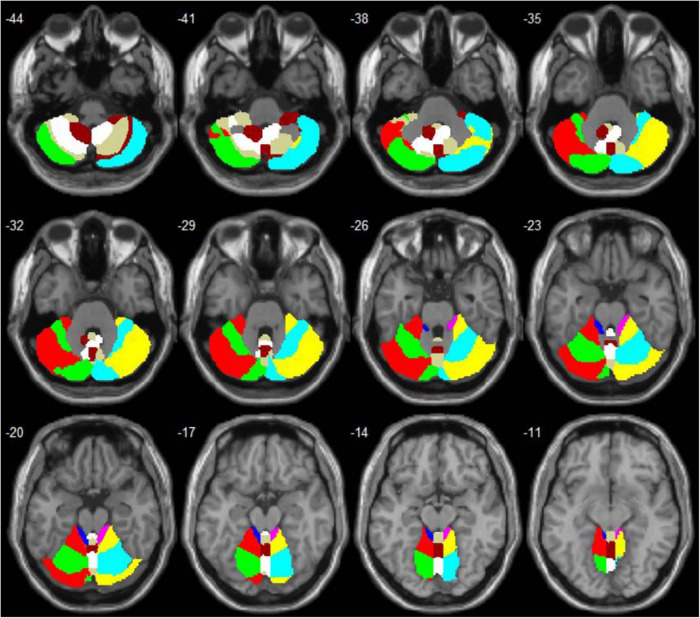
Anatomical automatic labeling (AAL) maps of the cerebellum and vermis subregions.

**FIGURE 2:**
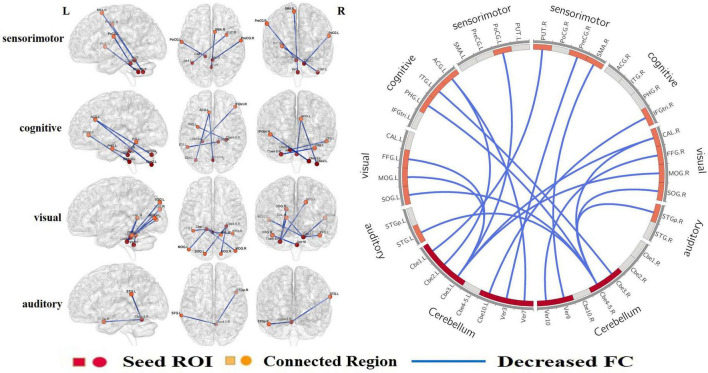
When all the cerebellar subregions were defined as ROIs, CTSCI patients showed significantly decreased FCs in the sensorimotor-, visual-, cognitive-, and auditory-related regions. ROI, regions of interests; CTSCI, complete thoracolumbar spinal cord injury; FC, functional connectivity; Cbe, cerebellum; Ver, vermis; PUT, Putamen; ACG, anterior cingulate gyrus; CAL, calcarine; FFG, fusiform; MOG, middle occipital gyrus; PoCG, postcentral gyrus; ITG, inferior temporal gyrus; SOG, supra-occipital gyrus; PHG, parahippocampal gyrus; STGp, superior temporal pole; STG, superior temporal gyrus; IFGtri, trigonometric inferior frontal gyrus; PreCG, precentral gyrus; SMA, supplementary motor area; R, right; L, left.

Compared to the HCs, patients with CTSCI showed slightly decreased GMV in Vermis_3 (*p* = 0.046, mean/std: CTSCI: 0.321 ± 0.036; HC: 0.351 ± 0.048). When all the cerebellar subregions (from the AAL116 template) were defined as ROIs (Figure 1), CTSCI patients showed significantly decreased FC in the sensorimotor-related areas (including the FC values between left cerebellum_1 and right putamen, left cerebellum_3 and left postcentral gyrus, vermis_3 and right precentral gyrus, vermis_9 and right supplementary motor area), visual-related regions (including the FC values between left cerebellum_3 and right calcarine, left fusiform, bilateral middle occipital gyrus; right cerebellum_4_5 and right calcarine, bilateral supra-occipital gyrus; vermis_10 and right fusiform), cognitive-related regions (including the FC values between left cerebellum_2 and left anterior cingulate gyrus, right cerebellum_3 and left inferior temporal gyrus, right cerebellum_4_5 and left parahippocampal gyrus, left cerebellum_10 and right trigonometric inferior frontal gyrus, vermis_7 and left anterior cingulate gyrus), and auditory-related regions (including the FC values between right cerebellum_4_5 and right superior temporal pole, left superior temporal gyrus) (cluster-level FWE correction with *p* < 0.05, Table 2 and Figure 2), compared to HCs.

**TABLE 2:** Regions of significant different connectivity between complete thoracolumbar spinal cord injury (CTSCI) patients and healthy controls (HCs).

ROIs	Brain regions	Cluster voxels	MNI coordinates (mm)			Maximum Z
			x	y	z	
Cbe1.L	PUT.R	47	33	6	3	−4.67
Cbe2.L	ACG.L	40	−6	24	27	−4.40
Cbe3.L	CAL.R	51	18	−51	6	−3.92
	FFG.L	91	−39	−42	−24	−5.96
	MOG.L	157	−48	−81	6	−5.23
	MOG.R	85	48	−84	3	−5.20
	PoCG.L	133	−60	−9	21	−4.68
Cbe3.R	ITG.L	97	−51	−48	−12	−4.92
Cbe4-5.R	CAL.R	76	24	−60	12	−4.44
	SOG.L	63	−15	−90	33	−4.77
	SOG.R	45	18	−90	21	−5.02
	PHG.L	63	−30	−6	−24	−5.07
	STGp.R	53	48	9	−24	−4.49
	STG.L	39	−66	−39	24	−4.53
Cbe10.L	IFGtri.R	102	48	36	0	−5.32
Ver3	PreCG.R	136	63	−6	42	−4.96
Ver7	ACG.L	54	0	18	30	−5.04
Ver9	SMA.R	51	9	12	63	−4.29
Ver10	FFG.R	43	42	−48	−24	−4.22

ROIs, regions of interest; CTSCI, complete thoracolumbar spinal cord injury; HC, healthy control; Cbe, cerebellum; Ver, vermis; PUT, Putamen; ACG, anterior cingulate gyrus; CAL, calcarine; FFG, fusiform; MOG, middle occipital gyrus; PoCG, postcentral gyrus; ITG, inferior temporal gyrus; SOG, supra-occipital gyrus; PHG, parahippocampal gyrus; STGp, superior temporal pole; STG, superior temporal gyrus; IFGtri, trigonometric inferior frontal gyrus; PreCG, precentral gyrus; SMA, supplementary motor area; R, right; L, left.

### Relationships between clinical variables and structure, functional connectivity values of cerebellar subregions in complete thoracolumbar spinal cord injury patients

The correlation analyses revealed that decreased FC values between the right cerebellum_4_5 and right supra-occipital gyrus weakly correlated with the visual analog scale (VAS) scores in CTSCI patients (*p* = 0.046, *r* = 0.521). No significant associations were detected between the other structure, FC values of cerebellum subregions and clinical features (ASIA scores and time since SCI) in CTSCI patients (*p* > 0.05).

### Support vector machine weight analysis on abnormal cerebellar functional features following complete thoracolumbar spinal cord injury patients

**FIGURE 3:**
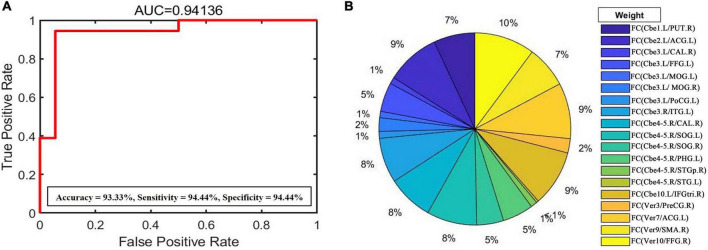
**(A)** Support vector machine (SVM) weight analysis was performed according to the predicted results to evaluate the impact of FC changes of each cerebellar subregion following CTSCI. The AUC value, accuracy, sensitivity, and specificity of this binary classification were 0.94, 93.33, 94.44, and 94.44%, respectively. **(B)** SVM weight analysis showed that the FC values between the vermis_10 and right fusiform gyrus had the greatest weight in the functional changes of CTSCI. FC, functional connectivity; CTSCI, complete thoracolumbar spinal cord injury; AUC, area under the curve; Cbe, cerebellum; Ver, vermis; PUT, Putamen; ACG, anterior cingulate gyrus; CAL, calcarine; FFG, fusiform; MOG, middle occipital gyrus; PoCG, postcentral gyrus; ITG, inferior temporal gyrus; SOG, supra-occipital gyrus; PHG, parahippocampal gyrus; STGp, superior temporal pole; STG, superior temporal gyrus; IFGtri, trigonometric inferior frontal gyrus; PreCG, precentral gyrus; SMA, supplementary motor area; R, right; L, left.

After the 10-fold cross-validation was completed, to evaluate the weight of abnormal cerebellar functional features in CTSCI, SVM weight analysis was performed according to the predicted results. The AUC value, accuracy, sensitivity, and specificity of this binary classification were 0.94, 93.33, 94.44, and 94.44%, respectively (Figure 3A). SVM weight analysis showed that the FC values between the vermis_10 and right fusiform gyrus had the greatest weight in the functional changes of CTSCI (Figure 3B).

## Discussion

The present study characterized structural changes in cerebellar subregions and abnormal FCs between the cerebellar subregions and the whole brain at resting-state in the CTSCI patients. In CTSCI patients, except for slightly decreased GMV in Vermis_3, the cerebellar subregions affect the function of the cerebral cortex through the change of alteration of FC, such as sensorimotor cortex, visual cortex, cognitive cortex, and auditory cortex), especially the FC changes between vermis 10 and right fusiform gyrus that play relatively important roles in affecting sensorimotor function.

### Brain structural and functional changes of cerebellar subregions in complete thoracolumbar spinal cord injury patients

The previous research on the FC changes of the whole brain mainly applies theA AL116 template (Tzourio-Mazoyer et al., 2002), which has a total of 116 brain regions, but only 90 regions belong to the cerebrum, which has been studied extensively, and the remaining 26 regions belong to the cerebellum, which has been seldom studied. The previous studies on the structure and function of SCI are mainly about the abnormal changes of the cerebral cortex, cerebellum is often considered to play an important role in the motion control, but a multidisciplinary study found that different cerebellum subregions play important roles in many fields, and changes in the cerebellar subregion after SCI have also been found in our previous studies. Therefore, exploring the neural mechanism of SCI from the perspective of cerebello-cerebral circuitry may find new clues. In the present study, CTSCI patients showed slightly structural atrophy in vermis_3, which is mainly related to sensorimotor function (Leicht and Schmidt, 1977). This may be due to the anatomical connection. Vermis_3 is a part of the spinocerebellar (Dietrichs, 2008); it is likely that this tract is affected by the injury.

As we known from cerebello-cortical circuit: The afferent fibers of cerebellum form the middle and lower cerebellar peduncles, transferring information mainly from the opposite cerebello-pontine nucleus and the inferior olivary nucleus to the new cerebellum, and the efferent fibers of cerebellum form the main body of the superior cerebellar peduncles, sending information from new cerebellum to the contralateral thalamus and cerebral cortex. From the view of the process, there is a close connection between the cerebellum and the cerebral functional cortex. Early studies believed that the main function of the cerebellum and vermis was motor control. In recent years, many neuroanatomy, neuroimaging, and clinical studies have shown that different sub-regions of the cerebellum and vermis participate in the different functions, such as learning, cognition, emotions, and behaviors (Reeber et al., 2013).

Spinal cord injury usually leads to severe sensory and motor deficits below the spinal cord lesion, owing to degenerative processes affecting efferent motor and afferent sensory pathways (Jurkiewicz et al., 2007; Wrigley et al., 2009). In the present study, we not only found decreased FC in the sensorimotor-related areas following CTSCI, which were consistent with the most previous studies (Zhu et al., 2015), but also found decreased FC in the visual-, cognitive-, and auditory-related regions. This can be explained by some previous research findings, which have shown that visual and/or memory information is involved in the regulation of sensorimotor function (Ramachandran and Altschuler, 2009). Our previous studies also found structural and functional changes in visual-related regions (Chen et al., 2018; Li et al., 2020). These anatomical and functional changes in the visual-related regions suggest that the visual-related regions may be involved in regulating sensorimotor function following CTSCI.

Additionally, some studies reported that up to 64% SCI patients have significant deficits in various cognitive domains (Davidoff et al., 1985, 1992). The poor cognitive performance in SCI patients may result from a deficit in cognitive output commands that rely on the feedback information received from the outside. Consistent with the previous results, we found decreased FC of cognition-related regions following CTSCI in this study. Motor imagery (MI) is a higher cognitive process; it triggers similar activations of brain regions as planning, preparation, and execution of movements (Decety and Jeannerod, 1996). In several neurological conditions, MI has been shown to have beneficial effects on rehabilitation (Schuster et al., 2011). MI has also been considered for rehabilitation of SCI patients, as it can facilitate functional recovery by allowing access to the motor network without the need for actual movement (Mulder, 2007).

This study also revealed a decreased FC of auditory-related regions following CTSCI, which may suggest that auditory-related regions could be also involved in regulating sensorimotor function. This is an interesting result and worthy of further study.

### Relationships between the functional connectivities of cerebellar subregions and clinical performances in complete thoracolumbar spinal cord injury patients

In subsequent correlation analysis, decreased FC values between the right cerebellum_4_5 and right supra-occipital gyrus revealed a weak positive correlation with the VAS scores in CTSCI patients. The supra-occipital gyrus belongs to the visual cortex, this result may suggest that functional changes in visual cortex are related to neuropathic pain, but the detailed results and possible mechanisms will be confirmed in our future studies of the task state experiment.

### Weight analysis based on functional connectivity features following complete thoracolumbar spinal cord injury

The SVM weight analysis showed that the FC values between the vermis_10 and right fusiform gyrus had the greatest weight in the functional changes of CTSCI. The fusiform belongs to the visual cortex; the previous studies have shown that visual tactile feedback and virtual reality (VR) training have become effective ways of motor rehabilitation training (Sayenko et al., 2010). In addition, some previous researchers found that with the additional visual information, patients would become more aware of the displacements in space and body’s orientation, to compensate for the loss of sensorimotor function (Cheng et al., 2004). This suggested that the FC change between vermis_10 and right fusiform gyrus might be used as key neural network targets of tDCS or brain–machine interface in CTSCI patients.

## Limitations

Some limitations of our study should be noted. First, the sample size was relatively small; a relatively large sample will be collected in the future to further verify our result. Second, our study is a cross-sectional study. Longitudinal studies are needed to perform to reveal the functional reorganization with disease progression, aim to search for different functional reorganization in different injury periods and develop targeted rehabilitation plan over time to improve maximize recovery. Finally, participants in this study did not receive visual stimulation, auditory- or cognition-related training, which will be applied to our follow-up study in the future.

## Conclusion

In conclusion, different degrees of structural and functional changes occurred in each subregion of cerebellum in CTSCI, and these changes affect the function of cerebral cortex through a variety of modes. Among these changes, FC changes between some cerebellar subregions and cerebral functional cortex (sensorimotor cortex, visual cortex, cognitive cortex, and auditory cortex), especially the FC change between vermis_10 and right fusiform gyrus might be used as key neural network targets in neuromodulation rehabilitation therapy such as tDCS or BCI, and relevant functional stimulation may contribute to the rehabilitation of sensorimotor function after CTSCI.

## Data availability statement

The datasets presented in this article are not readily available because it involves the privacy of the subjects. In addition, all the necessary information for the dataset has been included in our manuscript. Requests to access the datasets should be directed to WZ, 1013135963@qq.com.

## Ethics statement

The studies involving human participants were reviewed and approved by Ethics Committee of Xuanwu Hospital, Capital Medical University (Ethics No: [2020] 003). The patients/participants provided their written informed consent to participate in this study.

## Author contributions

WZ was responsible for study conception and design, the acquisition, analysis, and interpretation of data, drafting of the manuscript, final approval of the version of the manuscript to be published, and agreement to be accountable for all aspects of the work. LW, BY, QC, XL, XC, and WQ were responsible for data analysis, final approval of the version of the manuscript to be published, and agreement to be accountable for all aspects of the work. YH and JD were responsible for the clinical function assessment of patients. KL and JL were responsible for revising the manuscript, final approval of the version of the manuscript to be published, and agreement to be accountable for all aspects of the work. NC was responsible for study design, manuscript revision, final approval of the version of the manuscript to be published, and agreement to be accountable for all aspects of the work. All authors contributed to the article and approved the submitted version.

## Footnotes

1
http://www.fil.ion.ucl.ac.uk/spm/

## Abbreviations

CTSCI: complete thoracolumbar spinal cord injury
HC: healthy control
fMRI: functional magnetic resonance imaging
VBM: voxel-based morphometry
FC: functional connectivity
SVM: support vector machine
tDCS: transcranial direct current stimulation
VAS: visual analog scale
RS-fMRI: resting state functional magnetic resonance imaging
ASIA: American Spinal Cord Injury Association
EPI: echo-planar-imaging
DPARSF: data processing assistant for resting-state fMRI
SPM: statistical parametric mapping
FWE: family wise error
GM: gray matter
WM: white matter
CSF: cerebrospinal fluid
MNI: Montreal Neurological Institute
DARTEL: diffeomorphic anatomical registration through exponentiated lie algebra
FWHM: full-width at half-maximum
ROC: receiver operating characteristic
AUC: area under the curve
RAS: rhythmic auditory stimulation
MS: multiple sclerosis
PD: Parkinson’s disease
VR: virtual reality
MI: motor imagery.
